# The Sterilisation of Morphine Hydrochloride Solutions

**Published:** 1910-01-29

**Authors:** 


### Therapeutics.

### THE STERILISATION OF MORPHINE HYDROCHLORIDE SOLUTIONS.

It is remarkable how seldom any accidents result from hypodermic injection of morphine; nevertheless it is clearly wise to be sure that any solution of morphine that may be used for injection purposes is sterilised. It might be thought that all that was required to sterilise the solution would be to bring it to the boiling point and keep it at that for a few minutes. Such a procedure would sterilise the solution, it is true, but the matter is not quite so simple, because morphine itself is apt to undergo oxydation in the process of boiling, while the effects of the alkalinity of the glass in which the solution is boiled have to be considered.

By oxydation oxymorphine is formed, and this change is favoured by a minute trace of alkali; nearly all glass contains sufficient alkali for enough to be liberated under the influence of heat to change morphine into oxymorphine, and thus interfere with the activity of the drug. Consequently a trace of free acid in excess of any alkali that may be derived from glass should always be added to the solution, and whenever it is feasible the boiling should be carried out in a vessel whose surface is as little exposed to the free air as possible, seeing that the greater the access of oxygen the greater the liability of oxygenation.

